# Cross-linked versus conventional polyethylene for total knee arthroplasty: a meta-analysis

**DOI:** 10.1186/s13018-016-0374-1

**Published:** 2016-03-30

**Authors:** Bin-feng Yu, Guo-jing Yang, Wei-liang Wang, Lei Zhang, Xi-peng Lin

**Affiliations:** Department of Orthopedics, The Third Affiliated Hospital of Wenzhou Medical College, Wenzhou, 325200 People’s Republic of China

**Keywords:** Cross-linked, Polyethylene, Wear, Knee arthroplasty, Meta-analysis

## Abstract

**Background:**

Highly cross-linked polyethylene (HXLPE) has been reported as an effective material for decreasing polyethylene wear and osteolysis in total knee arthroplasty (TKA). Because no single study to date has been large enough to definitively determine the benefit of HXLPE in TKA, we conducted a meta-analysis to pool the results from randomized controlled trials (RCTs) and non-RCTs to make such a determination.

**Methods:**

Potential candidate articles were identified by searching the Cochrane Library, Medline (1966-2015.10), PubMed (1966-2015.10), Embase (1980-2015.10), ScienceDirect (1985-2015.10), and other databases. “Gray studies” were identified from the included articles’ reference lists. Pooled data were analyzed using RevMan 5.1.

**Results:**

Three RCTs and three non-RCTs were included in the meta-analysis. There were no significant differences between the groups in the total number of reoperations (*P* = 0.11), reoperations for prosthesis loosening (*P* = 0.08), radiolucent line (*P* = 0.20), osteolysis (*P* = 0.38), prosthesis loosening (*P* = 0.10), and mechanical failures related to the tibial polyethylene (*P* = 1.00). Similarly, no significant differences between the two groups were found in postoperative total knee score (*P* = 0.18) or functional score (*P* = 0.23).

**Conclusions:**

The meta-analysis showed that compared with conventional polyethylene, HXLPE did not improve the clinical and radiographic outcomes in mid-term follow-up after TKA. Additional high-quality multicenter prospective RCTs with good design, large study populations and long-term follow-up will be necessary to further clarify the effect of HXLPE in TKA.

## Background

Total knee arthroplasty (TKA) is a reliable and effective surgical treatment for end-stage knee arthritis. However, prosthesis loosening and osteolysis are major complications affecting the long-term survival of total knee prostheses [1, 2]. In clinical practice, younger and more active patients can experience early-onset osteolysis and extreme polyethylene wear [3]. Polyethylene wear has long been associated with osteolysis and prosthesis loosening in TKA [4–7]. Therefore, many studies have been conducted to introduce new designs and materials to reduce polyethylene wear and osteolysis with a goal of achieving better long-term results.

Mobile-bearing TKA was designed to reduce the peak loading stress and backside wear that was responsible for polyethylene wear in fixed-bearing designs [8, 9]. However, several high-quality studies indicated that the mobile-bearing designs did not result in better radiographic and clinical TKA outcomes. The superiority of mobile bearings was purely theoretical [10–13]. Highly cross-linked polyethylene (HXLPE), a modified form of conventional polyethylene, has a higher cross-link density achieved by irradiation. In recent years, HXLPE acetabular liners for total hip arthroplasty (THA) have successfully decreased linear wear and resulted in less osteolysis compared with conventional polyethylene (CP) [14, 15]. The superior performance of HXLPE in THA has led to its use in TKA. However, the process of cross-linking the polyethylene to improve wear had the negative effect of decreasing the mechanical properties of the highly cross-linked polyethylenes and adding free radicals to the polyethylene, which can lead to in vivo oxidation [16]. Additionally, one of the reasons attributed to the failure of HXLPE in TKA is that wear mechanisms in the knee are not exactly same as those in the hip [17]. Delamination, cracking, fatigue fracture, pitting, etc. are more common in TKA.

Some clinical studies reported inconsistent results in TKA using HXLPE [3, 17–21]. Consequently, the decision of whether to use HXLPE or conventional polyethylene remains controversial. To determine the effectiveness of HXLPE in primary TKA, there is a need for a comprehensive meta-analysis of large sample size randomized control trials (RCTs) and non-RCTs on the subject.

We hypothesized that, compared with conventional polyethylene, HXLPE would be associated with superior clinical and radiographic outcomes in primary TKA. The purpose of this study was to determine whether HXLPE improves postoperative outcomes compared with conventional polyethylene in TKA surgery.

## Methods

### Search strategy

Potential candidate articles were identified by searching the Cochrane Library, Medline (1966–2015.10), PubMed (1966–2015.10), Embase (1980–2015.10), and ScienceDirect (1985–2015.10) databases. “Gray studies” were identified from the reference lists of included articles so that relevant studies were not missed. No language was restricted. The key words “knee replacement OR arthroplasty,” “crosslink,” and “polyethylene” were used in combination with the Boolean operators AND or OR.

### Inclusion criteria

Studies were considered eligible for inclusion if they met the following criteria: (1) the patients underwent primary TKA; (2) the intervention was the use of HXLPE compared to CP (Table 1); (3) the outcomes included clinical outcomes, radiographic outcomes, complication, and revision reason; and (4) the study was a published or unpublished controlled clinical trial.

**Table 1:** Characteristics of included studies

Study	Study design	Group	Simple size	Age (year)	Gender (M/F)	BMI	Manufacture	Devices information	Follow-up	Lost follow-up
Hodrick et al. 2008 [3]	CCT	XLPE	100	67 ± 12	42/58	NS	Compression-molded polyethylene sterilized with ethylene oxide gas preheated at 125°m and irradiated with a dose 9.5 Mrad (95 kGy) through an electron beam.	Natural knee II system (Zimmer)	Mean 75 months	18
		CP	100	70 ± 12	40/60	NS	Compression-molded polyethylene, which was gamma-irradiated in nitrogen.		Mean 82 months	17
Minoda et al. 2009 [21]	CCT	XLPE	89	70.3 ± 8.9	19/70	NS	GUR 1050 UHMWPE bar cross-linked by subjection of 65 kGy of electron beam with thermal treatment above melting point, and were sterilized by gas plasma.	NexGen CR. Zimmer)	2 years	0
		CP	113	71.1 ± 7.3	20/93	NS	Net-shape molding of GUR 1050 resin and sterilized by gamma radiation in nitrogen at a dose of 37 kGy.			0
Lachiewicz and Soileau 2015 [19]	RCT	XLPE	110	68 ± 10	35/75	31	Electron beam, 6.5 CGy irradiated in nitrogen, remelted to quench the free radicals; then sterilized by ethylene oxide	Zimmer, Inc, Warsaw, IN, USA	Minimum 2 years	14
		CP	122	70 ± 10	37/85	31	Gamma-irradiated in nitrogen			21
Meneghini et al. 2015 [20]	PCT	XLPE	64	63.8	18/29	31.5	GUR 1020 UHMWPE and cross-linking was performed with a cycled process of gamma-irradiation at 30 kGy followed by annealing at 130 °C for 8 h, which was repeated 3 times sequentially	Posterior-substituting single-radius design (Triathlon; Stryker, Mahwah, NJ)	5.2 years (range, 4.3–5.8 years).	10
		CP	50	67.3	12/25	31.1	Compression-molded GU 1020 UHMWPE, packed in nitrogen and gamma-irradiated at 30 kGy.		5.5 years (range, 4.8–7.4 years)	
Kim and Park 2014 [17]	RCT	XLPE	308	60.3 ± 4.3	20/288	29.1	Machined from GUR1050 resin bar.	NexGen Legacy Posterior-stabilized (LPS)-Flex total knee prosthesis (Zimmer,Warsaw, Indiana)	59 years (range, 5–6.8 years)	0
		CP	308	60.3 ± 4.3	20/288	29.1	Machined from GUR1050 resin bar, cross-linked by a 65-kGy electron beam			0
Kindsfater et al. 2015 [18]	RCT	XLPE	477	66.4 ± 8.5	35.4 %/64.6 %	32.6	NS	P.F.C. Sigma fixed-bearing knees (DePuy Synthes Joint Reconstruction, Warsaw, IN).	5-year	298
		CP	449	66.3 ± 8.5	35.4 %/64.6 %	33.2	NS			260

### Exclusive criteria

We excluded articles that (1) duplicate reports of earlier trials or post hoc analyses of data; (2) articles without an available full-text version; (3) no available outcome data; and (4) non-English-language articles.

### Selection criteria

Two reviewers independently conducted the search process. Subsequently, the full text of the studies that potentially met the inclusion criteria were read, and the literature was reviewed to determine the final inclusion. Disagreements were resolved though consultation with a third reviewer.

### Quality assessment

Quality assessment of the randomized trials was conducted using a modification of the generic evaluation tool used by the Cochrane Bone, Joint and Muscle Trauma Group [22]; the index for non-randomized studies (MINORS) form was used for the non-randomized clinical trials [23]. The methodological quality of each trial was scored from 0 to 24. Disagreements were resolved by consensus or consultation with the senior reviewer.

### Data extraction

Two researchers independently extracted the data from the included articles. In cases of incomplete data, the corresponding author was contacted to provide the missing details. The following information was extracted from the articles: first author name, publication year, intervening measures, comparable baseline, sample size, and outcome measures. Other relevant parameters were also extracted from individual studies.

### Data analysis and statistical methods

The pooled data were analyzed using RevMan 5.1 software (The Cochrane Collaboration, Oxford, UK). Heterogeneity was estimated based on the values of *P* and *I*^2^ using a standard chi-square test. When *I*^2^ > 50 %, *P* < 0.1 was considered to be significant heterogeneity; in this case, a random-effects model was applied for data analysis. A fixed-effects model was used when no significant heterogeneity was found. In cases of significant heterogeneity, subgroup analysis was performed to identify the sources of the heterogeneity. For continuous data, mean differences (MDs) and 95 % confidence intervals (CIs) were calculated. Risk differences (RD) and 95 % CIs were calculated for dichotomous data.

## Results

### Search results

**Fig. 1:**
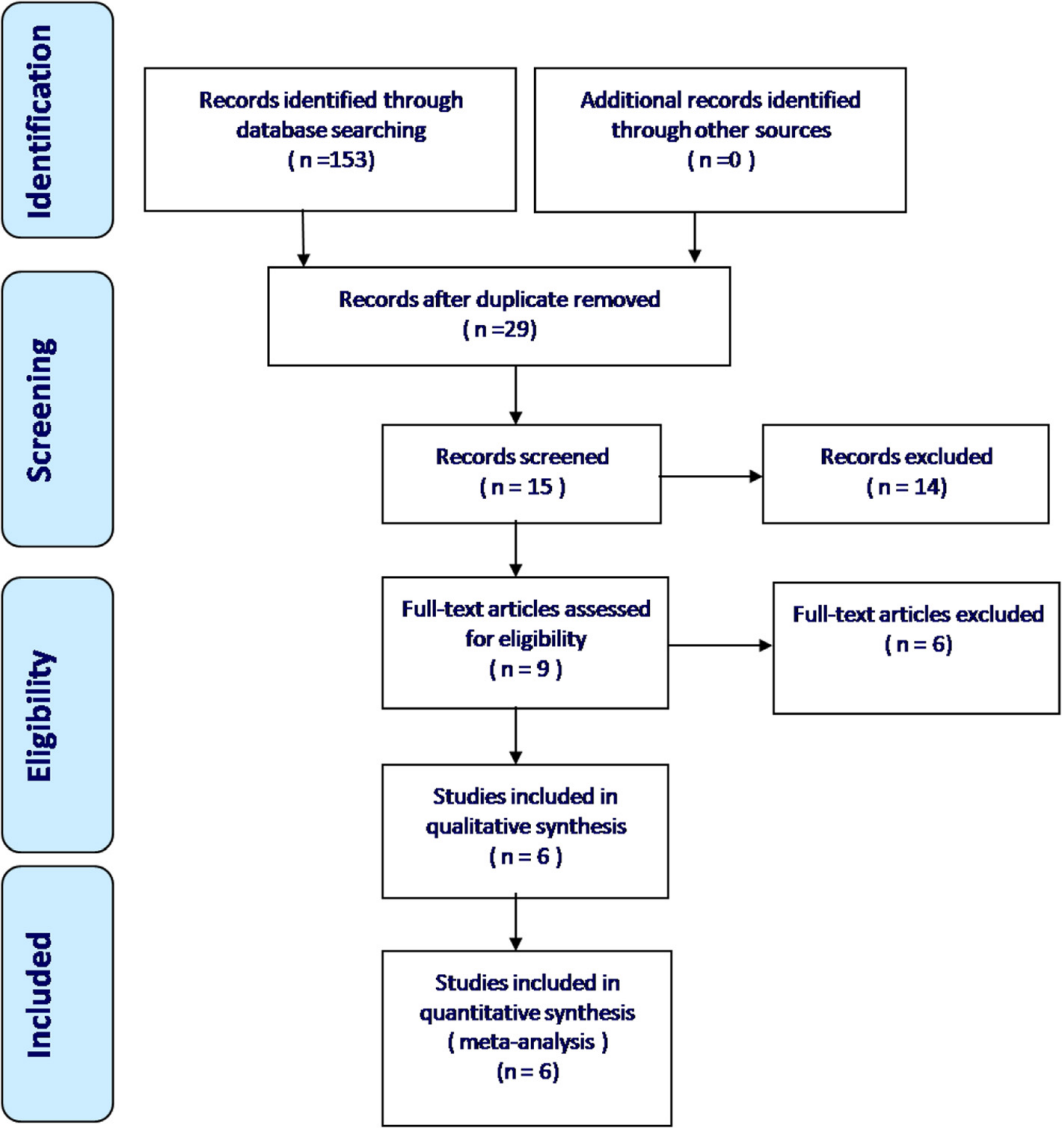
Flowchart of the study selection process

A total of 153 potentially relevant studies were identified. No additional studies were identified after review of the references. After browsing the titles and abstracts and reviewing the full text, three non-RCTs and three RCTs were eligible for data extraction and were included in the meta-analysis. The articles were published between 2008 and 2015, and they each specified detailed inclusion criteria. The search process is illustrated in Fig. 1.

### Risk of bias assessment

**Fig. 2:**
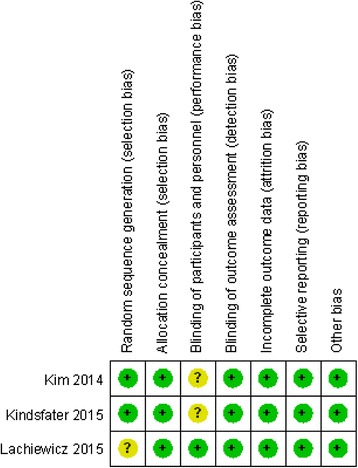
Risk of bias summary of randomized control trials

The assessment of RCT quality was based on the Cochrane Handbook for Systematic Review of Interventions (Fig. 2). All RCTs stated clear inclusion criteria, and two of them provided a methodology of randomization; they stated that the method of randomization was the closed envelope technique. A blinding assessor was provided in all RCTs. One of the RCTs attempted to blind the participants or the surgeon. No studies had unclear bias due to incomplete outcome data or selective outcome reporting. For the non-RCTs [13–16], the MINORS score was 16–18 for the retrospectively controlled trials. The methodological quality assessment is illustrated in Table 2.

**Table 2:** Quality assessment for non-randomized trials

Quality assessment for non-randomized trials	Hodrick et al. 2008 [3]	Minoda et al. 2009 [21]	Meneghini et al. 2015 [20]
A clearly stated aim	2	2	2
Inclusion of consecutive patients	2	2	2
Prospective data collection	1	1	2
Endpoints appropriate to the aim of the study	1	1	1
Unbiased assessment of the study endpoint	1	1	1
A follow-up period appropriate to the aims of study	2	2	2
Less than 5 % loss to follow-up	0	2	0
Prospective calculation of the sample size	0	0	0
An adequate control group	2	2	2
Contemporary groups	1	1	1
Baseline equivalence of groups	2	2	2
Adequate statistical analyses	2	2	2
Total score	16	18	17

### Study characteristics

The demographic characteristics and details of the included studies are summarized in Table 1. Only patients diagnosed with end-stage arthritis were enrolled. Statistically similar baseline characteristics were observed between two groups. The experimental group used HXLPE, while the control group used CP. The design of the knee prosthesis varied among the different studies, but all used a medial parapatellar surgical approach. Standard rehabilitation methods were used in all studies.

### Outcomes for meta-analysis

#### Total reoperations

**Fig. 3:**
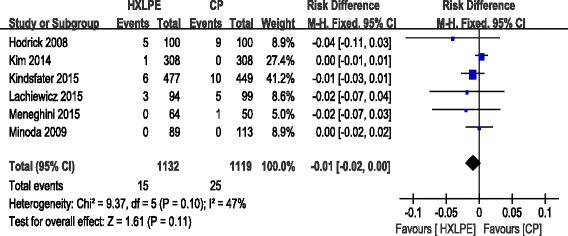
Forest plot diagram showing total reoperations between two groups

All of the studies reported the overall incidence of reoperation. There was no significant heterogeneity among the studies (*χ*^2^ = 9.37, df = 5, *I*^2^ = 47 %, *P* = 0.10), so a fixed-effects model was utilized. Pooling resulted in no significant difference between the two groups (RD = −0.01, 95 % CI −0.02 to 0.00, *P* = 0.11; Fig. 3).

#### Reoperation for prosthesis loosening

**Fig. 4:**
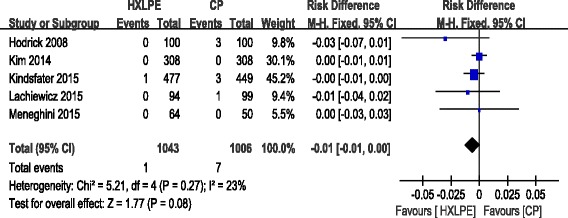
Forest plot diagram showing reoperation for prosthesis loosening between two groups

Reoperation for prosthesis loosening was reported in five studies. No significant heterogeneity was identified, so a fixed-effects model was applied (*χ*^2^ = 5.21, df = 4, *I*^2^ = 23 %, *P* = 0.27). There was no significant difference between the two groups (RD = −0.01, 95 % CI −0.01 to 0.00, *P* = 0.08; Fig. 4).

#### Radiolucent line

**Fig. 5:**
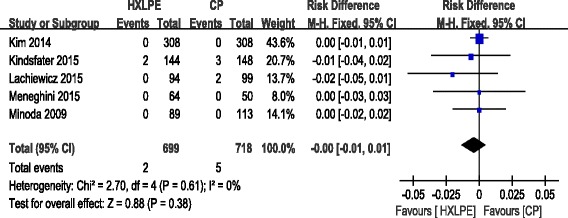
Forest plot diagram showing radiolucent line between two groups

A radiolucent line was reported in four studies. No significant heterogeneity was found, so a random-effects model was applied (*χ*^2^ = 13.81, df = 3, *I*^2^ = 78 %, *P* = 0.003). There was no significant difference between the two groups (RD = −0.05, 95 % CI −0.13 to 0.04, *P* = 0.20; Fig. 5).

#### Osteolysis

Osteolysis was reported in five studies. No significant heterogeneity was found, so a fixed-effects model was used (*χ*^2^ = 2.70, df = 4, *I*^2^ = 0 %, *P* = 0.61). There was no significant difference between the two groups (RD = −0.0, 95 % CI −0.01 to 0.01, *P* = 0.38).

#### Prosthesis loosening

Four articles reported the incidence of prosthesis loosening after surgery. A fixed-effects model was used because of low significant heterogeneity (*χ*^2^ = 6.06, df = 3, *I*^2^ = 51 %, *P* = 0.11). No significant difference was observed between the groups (RD = −0.01, 95 % CI −0.01 to 0.00, *P* = 0.10).

#### Mechanical failures related to the tibial polyethylene

Three studies described mechanical failures related to the tibial polyethylene. No significant heterogeneity was shown between pooling results; thus, a fixed-effects model was utilized (*χ*^2^ = 0.0, df = 2, *I*^2^ = 0 %, *P* = 1.00). A significant difference was observed between the groups in terms of length of hospital stay (RD = 0.00, 95 % CI −0.01 to 0.01, *P* = 1.00).

#### Postoperative total knee score

The postoperative total knee score was reported in three studies. No significant heterogeneity was found, so a fixed-effects model was used (*χ*^2^ = 0.28, df = 2, *I*^2^ = 0 %, *P* = 0.87). No significant difference was observed between the groups (MD = −0.61, 95 % CI −1.49 to 0.28, *P* = 0.18).

#### Postoperative functional score

Three studies reported a postoperative functional score. No significant heterogeneity was shown between the pooled results, and, thus, a fixed-effects model was utilized (*χ*^2^ = 0.36, df = 2, *I*^2^ = 0 %, *P* = 0.84). There was no significant difference between the groups in terms of postoperative functional score (MD = 1.61, 95 % CI −1.02 to 4.25, *P* = 0.23).

## Discussion

The most important finding of this meta-analysis was that, compared with CP, HXLPE did not decrease the incidence of a postoperative radiolucent line, osteolysis, prosthesis loosening, or reoperation after TKA. XLPE did not improve postoperative clinical outcomes in American Knee Society Knee score based on the results; XLPE showed no advantage over CP in TKA.

Polyethylene wear and subsequent osteolysis in total joint arthroplasty could result in patient dissatisfaction and short survivorship of the prosthetic implant, which increases the medical burden. HXLPE successfully decreased polyethylene wear, osteolysis, and revision rates in THA; however, the wear mechanisms and particle debris occurring in TKA differ substantially from those in THA [17]. In vitro studies showed that HXLPE positively affected the wear characteristics in TKA [24, 25]; as a result, many orthopedists adopted HXLPE to solve wear and related problems in TKA. To date, the potential benefits of HXLPE have not been confirmed in the published research. Although a few reviews of the biomechanical and clinical evidence for HXLPE in TKA have been published, they did not extract data for further quantitative analysis [26, 27]. To our knowledge, this is the first quantitative large sample size meta-analysis to study HXLPE in TKA by only including studies with appropriate control and study groups.

Wear of the polyethylene liner generates particles that induce osteolysis. Studies have reported that HXLPE generates smaller particles than CP [5]. These smaller particles may be more biologically active and theoretically could lead to more osteolysis [28–30]. The frequency of periprosthetic osteolysis after TKA has been reported to be 5 to 20 % over a follow-up period of 5 to 15 years [7, 31, 32]. In our meta-analysis of five studies, no significant difference was found in the incidence of osteolysis, which was 0.29 % in the HXLPE group and 0.69 % in the CP group. The incidence of osteolysis was lower than that reported previously, probably because the follow-up periods of the studies included in the meta-analysis (2–6 years) were relatively shorter than in previous reports. Increased osteolysis may be observed in patients over longer-term follow-up.

The reasons for reoperation after TKA included polyethylene wear, prosthesis loosening, periprosthetic infection, mal-alignment, instability, arthrofibrosis, and periprosthetic fracture [33, 34]. In our meta-analysis, no significant difference was observed in the incidence of reoperation between groups. For HXLPE, we are more concerned about reoperation performed for prosthesis loosening. The results of the meta-analysis showed that, compared with CP, HXLPE did not decrease the incidence of prosthesis loosening and related reoperations with short- or middle-term follow-up. It is important to note that prosthesis loosening and related reoperations would increase with longer-term follow-up.

Postoperative knee function is another important index to assess the effect of HXLPE in TKA. Because different scoring systems were used, we counted the scoring systems as completely as possible. The pooled results indicated that HXLPE did not result in improved KSS knee and KSS function. In Kim’s study, the two groups did not differ significantly in terms of postoperative WOMAC scores, patient satisfaction assessed on a visual analog scale, and UCLA activity score [17]. Two of the included studies also found no significant difference in ROM between the two groups [3, 21]. We concluded that HXLPE did not result in better clinical outcomes than CP.

HXLPE has also been associated with weak mechanical properties, including strength and fatigue resistance and even reduced fracture toughness [35, 36]. Ries et al. believed that the use of HXLPE in TKA may contribute to mechanical failure and concluded that it should not be used in TKA [37, 38]. A case was reported in the literature of XLPE post fracture due to the higher local stresses in the posterior-stabilized designs [39]. Of the six included studies, three indicated that there were no mechanical failures related to the tibial polyethylene in either group. Similarly, the pooled results showed no significant difference. Therefore, we concluded that HXLPE was as safe as CP in TKA.

There are some potential limitations in this meta-analysis. (1) Only three RCTs and three non-RCTs were identified, with small sample sizes and relatively short-term follow-up. (2) Methodological weaknesses existed in all of the studies. (3) Some of the data, such as ROM, are inappropriate for meta-analysis.

## Conclusions

In conclusion, this meta-analysis showed that, compared with CP, HXLPE did not improve the clinical and radiographic outcomes in mid-term follow-up after TKA. Additional high-quality multicenter prospective RCTs with good design, large study populations, and long-term follow-up will be necessary to further clarify the effect of HXLPE in TKA.

## References

[1] Berry DJ (2004). Recognizing and identifying osteolysis around total knee arthroplasty. Instr Course Lect.

[2] Wang S, Xia J, Wei Y, Wu J, Huang G (2014). Effect of the knee position during wound closure after total knee arthroplasty on early knee function recovery. J Orthop Surg Res.

[3] Hodrick JT, Severson EP, McAlister DS, Dahl B, Hofmann AA (2008). Highly crosslinked polyethylene is safe for use in total knee arthroplasty. Clin Orthop Relat Res.

[4] DeHeer DH, Engels JA, DeVries AS, Knapp RH, Beebe JD (2001). In situ complement activation by polyethylene wear debris. J Biomed Mater Res.

[5] Fisher J, McEwen HM, Tipper JL, Galvin AL, Ingram J, Kamali A, et al. Wear, debris, and biologic activity of cross-linked polyethylene in the knee: benefits and potential concerns. Clin Orthop Relat Res. 2004;428:114–9. 10.1097/01.blo.0000148783.20469.4c 15534530

[6] Green TR, Fisher J, Matthews JB, Stone MH, Ingham E (2000). Effect of size and dose on bone resorption activity of macrophages by in vitro clinically relevant ultra high molecular weight polyethylene particles. J Biomed Mater Res.

[7] Naudie DD, Ammeen DJ, Engh GA, Rorabeck CH (2007). Wear and osteolysis around total knee arthroplasty. J Am Acad Orthop Surg.

[8] Dalury DF, Barrett WP, Mason JB, Goldstein WM, Murphy JA, Roche MW (2008). Midterm survival of a contemporary modular total knee replacement: a multicentre study of 1970 knees. J Bone Joint Surg Br.

[9] Dixon MC, Brown RR, Parsch D, Scott RD (2005). Modular fixed-bearing total knee arthroplasty with retention of the posterior cruciate ligament. A study of patients followed for a minimum of fifteen years. J Bone Joint Surg Am.

[10] Kim YH, Kim DY, Kim JS (2007). Simultaneous mobile- and fixed-bearing total knee replacement in the same patients. A prospective comparison of mid-term outcomes using a similar design of prosthesis. J Bone Joint Surg Br.

[11] Kim YH, Yoon SH, Kim JS (2007). The long-term results of simultaneous fixed-bearing and mobile-bearing total knee replacements performed in the same patient. J Bone Joint Surg Br.

[12] Poirier N, Graf P, Dubrana F (2015). Mobile-bearing versus fixed-bearing total knee implants. Results of a series of 100 randomised cases after 9 years follow-up. Orthop Traumatol Surg Res.

[13] Tjornild M, Soballe K, Hansen PM, Holm C, Stilling M (2015). Mobile- vs. fixed-bearing total knee replacement. Acta Orthop.

[14] Bragdon CR, Doerner M, Martell J, Jarrett B, Palm H, Malchau H (2013). The 2012 John Charnley Award: clinical multicenter studies of the wear performance of highly crosslinked remelted polyethylene in THA. Clin Orthop Relat Res.

[15] Mall NA, Nunley RM, Zhu JJ, Maloney WJ, Barrack RL, Clohisy JC (2011). The incidence of acetabular osteolysis in young patients with conventional versus highly crosslinked polyethylene. Clin Orthop Relat Res.

[16] Muratoglu OK, Bragdon CR, O'Connor DO, Jasty M, Harris WH (2001). A novel method of cross-linking ultra-high-molecular-weight polyethylene to improve wear, reduce oxidation, and retain mechanical properties. Recipient of the 1999 HAP Paul Award. J Arthroplasty.

[17] Kim YH, Park JW (2014). Comparison of highly cross-linked and conventional polyethylene in posterior cruciate-substituting total knee arthroplasty in the same patients. J Bone Joint Surg Am.

[18] Kindsfater KA, Pomeroy D, Clark CR, Gruen TA, Murphy J, Himden S (2015). In vivo performance of moderately crosslinked, thermally treated polyethylene in a prospective randomized controlled primary total knee arthroplasty trial. J Arthroplasty.

[19] Lachiewicz PF, Soileau ES (2016). Is there a benefit to highly crosslinked polyethylene in posterior-stabilized total knee arthroplasty? a randomized trial. Clin Orthop Relat Res.

[20] Meneghini RM, Lovro LR, Smits SA, Ireland PH (2015). Highly cross-linked versus conventional polyethylene in posterior-stabilized total knee arthroplasty at a mean 5-year follow-up. J Arthroplasty.

[21] Minoda Y, Aihara M, Sakawa A, Fukuoka S, Hayakawa K, Tomita M, et al. Comparison between highly cross-linked and conventional polyethylene in total knee arthroplasty. Knee. 2009;16(5):348–51. 10.1016/j.knee.2009.01.005 19268598

[22] Handoll HH, Gillespie WJ, Gillespie LD, Madhok R (2008). The Cochrane Collaboration: a leading role in producing reliable evidence to inform healthcare decisions in musculoskeletal trauma and disorders. Indian J Orthop.

[23] Slim K, Nini E, Forestier D, Kwiatkowski F, Panis Y, Chipponi J (2003). Methodological index for non-randomized studies (minors): development and validation of a new instrument. ANZ J Surg.

[24] Muratoglu OK, Bragdon CR, Jasty M, O'Connor DO, Von Knoch RS, Harris WH (2004). Knee-simulator testing of conventional and cross-linked polyethylene tibial inserts. J Arthroplasty.

[25] Muratoglu OK, Bragdon CR, O'Connor DO, Perinchief RS, Jasty M, Harris WH (2002). Aggressive wear testing of a cross-linked polyethylene in total knee arthroplasty. Clin Orthop Relat Res.

[26] Renner L, Faschingbauer M, Boettner F (2015). Is there a rationale to use highly cross-linked polyethylene in posterior-stabilized total knee arthroplasty?. Ann Transl Med.

[27] Sakellariou VI, Sculco P, Poultsides L, Wright T, Sculco TP (2013). Highly cross-linked polyethylene may not have an advantage in total knee arthroplasty. HSS J.

[28] Engh GA, Ammeen DJ (2004). Epidemiology of osteolysis: backside implant wear. Instr Course Lect.

[29] Huang CH, Ho FY, Ma HM, Yang CT, Liau JJ, Kao HC, et al. Particle size and morphology of UHMWPE wear debris in failed total knee arthroplasties—a comparison between mobile bearing and fixed bearing knees. J Orthop Res. 2002;20(5):1038–41. 10.1016/S0736-0266(02)00015-3 12382971

[30] Short A, Gill HS, Marks B, Waite JC, Kellett CF, Price AJ, et al. A novel method for in vivo knee prosthesis wear measurement. J Biomech. 2005;38(2):315–22. 10.1016/j.jbiomech.2004.02.023 15598459

[31] Lachiewicz PF, Soileau ES (2009). Fifteen-year survival and osteolysis associated with a modular posterior stabilized knee replacement. A concise follow-up of a previous report. J Bone Joint Surg Am.

[32] Lachiewicz PF, Soileau ES (2014). Fixation, survival and osteolysis with a modern posterior-stabilized total knee arthroplasty. J Arthroplasty.

[33] Indelli PF, Marcucci M, Graceffa A, Charlton S, Latella L (2014). Primary posterior stabilized total knee arthroplasty: analysis of different instrumentation. J Orthop Surg Res.

[34] Piepers MJ, van Hove RP, van den Bekerom MP, Nolte PA (2014). Do refinements to original designs improve outcome of total knee replacement? A retrospective cohort study. J Orthop Surg Res.

[35] Bradford L, Baker D, Ries MD, Pruitt LA (2004). Fatigue crack propagation resistance of highly crosslinked polyethylene. Clin Orthop Relat Res.

[36] Naudie DD, Rorabeck CH (2004). Sources of osteolysis around total knee arthroplasty: wear of the bearing surface. Instr Course Lect.

[37] Ries MD (2005). Highly cross-linked polyethylene: the debate is over—in opposition. J Arthroplasty.

[38] Ries MD, Pruitt L (2005). Effect of cross-linking on the microstructure and mechanical properties of ultra-high molecular weight polyethylene. Clin Orthop Relat Res.

[39] Jung KA, Lee SC, Hwang SH, Kim SM (2008). Fracture of a second-generation highly cross-linked UHMWPE tibial post in a posterior-stabilized scorpio knee system. Orthopedics.

